# FECTS: A Facial Emotion Cognition and Training System for Chinese Children with Autism Spectrum Disorder

**DOI:** 10.1155/2022/9213526

**Published:** 2022-04-27

**Authors:** Guobin Wan, Fuhao Deng, Zijian Jiang, Sifan Song, Di Hu, Lifu Chen, Haibo Wang, Miaochun Li, Gong Chen, Ting Yan, Jionglong Su, Jiaming Zhang

**Affiliations:** ^1^Shenzhen Maternal and Child Health Hospital, Shenzhen 518000, China; ^2^Shenzhen Institute of Artificial Intelligence and Robotics for Society, Shenzhen 518172, China; ^3^Department of Mathematical Sciences, Xi'an Jiaotong-Liverpool University, Suzhou 215123, China; ^4^University of Maryland, Robert H.Smith School of Business, College Park, MA, USA; ^5^DoGoodly International Education Center (Shenzhen) Co., Ltd. and Smart Children Education Center, Shenzhen 518219, China; ^6^School of Information and Control Engineering, China University of Mining and Technology, Xuzhou 221116, China; ^7^Department of Information Management and Information System, Guangdong Pharmaceutical University, Zhongshan 511436, China; ^8^Sunwoda Electronic Co., Ltd, Shiyan Street, Bao'an District, Shenzhen 518000, China; ^9^Shenzhen Key Laboratory for Molecular Biology of Neural Development, Guangdong Provincial Key Laboratory of Brain Connectome and Behavior, CAS Key Laboratory of Brain Connectome and Manipulation, The Brain Cognition and Brain Disease Institute, Shenzhen Institutes of Advanced Technology, Chinese Academy of Sciences, Shenzhen-Hong Kong Institute of Brain Science, Shenzhen, Guangdong 518055, China; ^10^School of AI and Advanced Computing, XJTLU Entrepreneur College (Taicang), Xi'an Jiaotong-Liverpool University, Suzhou, Jiangsu 215123, China; ^11^Institute of Robotics and Intelligent Manufacturing, The Chinese University of Hong Kong (Shenzhen), Shenzhen 518172, China

## Abstract

Traditional training methods such as card teaching, assistive technologies (e.g., augmented reality/virtual reality games and smartphone apps), DVDs, human-computer interactions, and human-robot interactions are widely applied in autistic rehabilitation training in recent years. In this article, we propose a novel framework for human-computer/robot interaction and introduce a preliminary intervention study for improving the emotion recognition of Chinese children with an autism spectrum disorder. The core of the framework is the Facial Emotion Cognition and Training System (FECTS, including six tasks to train children with ASD to match, infer, and imitate the facial expressions of happiness, sadness, fear, and anger) based on Simon Baron-Cohen's E-S (empathizing-systemizing) theory. Our system may be implemented on PCs, smartphones, mobile devices such as PADs, and robots. The training record (e.g., a tracked record of emotion imitation) of the Chinese autistic children interacting with the device implemented using our FECTS will be uploaded and stored in the database of a cloud-based evaluation system. Therapists and parents can access the analysis of the emotion learning progress of these autistic children using the cloud-based evaluation system. Deep-learning algorithms of facial expressions recognition and attention analysis will be deployed in the back end (e.g., devices such as a PC, a robotic system, or a cloud system) implementing our FECTS, which can perform real-time tracking of the imitation quality and attention of the autistic children during the expression imitation phase. In this preliminary clinical study, a total of 10 Chinese autistic children aged 3–8 are recruited, and each of them received a single 20-minute training session every day for four consecutive days. Our preliminary results validated the feasibility of the developed FECTS and the effectiveness of our algorithms based on Chinese children with an autism spectrum disorder. To verify that our FECTS can be further adapted to children from other countries, children with different cultural/sociological/linguistic contexts should be recruited in future studies.

## 1. Introduction

ASD (autism spectrum disorder) is characterized by traits such as impaired social communication, restrictive and repetitive behaviors, as well as narrow interests (Diagnostic and Statistical Manual of Mental Disorders 5th Edition: DSM 5). Difficulty in understanding the emotional and mental states of others plays a major role in the social and communicative characteristics of ASC (autism spectrum conditions) [1], and individuals with ASC exhibit delays in the development of the ability to recognize and discriminate emotional expressions [2]. It is estimated that approximately 52 million people worldwide live with ASD [3]. Furthermore, it is estimated that over 10 million people are living with ASD and over 2 million with childhood autism in mainland China [4]. According to a report in Modern Education News in Chinese [5], there is a lack of qualified autistic professionals in rehabilitation training for autistic people in China.

To address the issue of an increasing population of autistic people worldwide every year, it is necessary for researchers in all fields to work with social scientists, cognitive scientists, neuroscience scientists, developmental scientists, and domain experts to understand the mechanism of autism. There are certain kinds of cognitive theories in the psychological research of autistic mechanisms such as the mind-blindness (MB) theory [6], the weak central coherence (WCC) theory [7], the executive dysfunction (ED) theory [8–10], and the empathy-systemizing (E-S) theory [11]. According to Baron-Cohen [11], the E-S theory seems more applicable and better suited to explain the whole set of features characterizing ASC than the MB, WCC, or ED theory.

The E-S theory is a two-factor theory that can the social and communication difficulties by using below-average empathy, as well as explain the narrow interests, repetitive behavior, and resistance to change/need for sameness by using average or even above-average systemizing [11]. In this theory, empathy consists of two components, that is, the cognitive component that identifies someone's (or your own) mental state and the responsive component that reacts to another person's thoughts and feelings emotionally and appropriately. In contrast, systemizing is defined as the drive to analyze or construct systems that follow rules, that is, the drive to identify the rules that govern the system, in order to predict how that system will behave. To explain why the E-S theory can account for the preference of people with ASC for systems that change in highly lawful or predictable ways and why they become disabled when faced with systems characterized by less lawful change and their “need for sameness” or “resistance to change,” Baron-Cohen introduces the concept of ***truth*** as precise, reliable, consistent, or lawful patterns or structure in data and views people with ASC as strongly driven to discover the ***truth*** [11]. Baron-Cohen argues that the autistic mind in search of “***truth***” can explain why people with ASC may struggle with empathy and be less interested in topics such as pure fiction, pretense, or deception such as emotions and mental states that are not, and never will be, ***truth***-oriented [11]. Furthermore, Baron-Cohen argued that there may be a degree of trade-off between empathy (*E*) and systemizing (S) such that some people with ASC or with Asperger syndrome may have difficulties with empathy that cannot easily be systemized but they may have superior skill in systemizing.

To improve the effectiveness of rehabilitation training and the diversification of treatment, as well as to alleviate the shortage of medical personnel in autistic rehabilitation institutions, in addition to the traditional training methods such as card teaching, assistive technologies (e.g., augmented reality/virtual reality games and smartphone apps), DVDs, human-computer interactions, and human-robot interactions have been widely applied in autistic rehabilitation training in recent years. For instance, according to Autism Speaks [12], augmentative and alternative communication (AAC) can benefit people with autism of all ages by promoting independence, expanding communication, and increasing social interactions. Augmented reality is applied for assistive and learning objectives, such as detecting words from labial movements (i.e., automated lip detection) [13] that allows a better understanding of autistic people; virtual reality is used for prosthetic training [14] or neural telerehabilitation of patients with stroke [15]; this can also play a role in some training tasks for people with Autism.

Card teaching only, or card teaching combined with other materials such as textbooks or socially interactive robots, has demonstrated their rehabilitation effect in training autistic children for emotion recognition. To improve the social communication abilities of autistic children in Hong Kong, Fuk Chuen Ho and his colleagues in the Hong Kong Institute of Education and the Education Bureau published a textbook series of “Theory of Mind I-IV: Teaching Children with Autism Spectrum Disorders Emotions (in Chinese)” [16–19] to guide teachers and parents, in which storytelling, card teaching, role-playing, multimedia teaching, teaching software are utilized as multi-teaching measures. The textbook series are designed based on the theories and contents in “Teaching Children with Autism to Mind-read: A Practical Guide for Teachers and Parents” [20] that aims to provide practical easy-to-follow graded teaching guidelines for helping autistic children improve their understanding of beliefs, emotions, and pretense by applying the “theory of mind” or mind-blindness (MB) theory. A research group at the MIT has pioneered the use of card teaching combined with a socially interactive robot to teach autistic children in Japan and Serbia to match the facial expressions on cards with the facial expressions of the robot and to recognize and imitate the expressions of the robot [21, 22].

For smartphone apps, according to [23], only a small portion of nearly 700 mobile device apps listed on the “Autism Apps” section on Autism Speaks website [24] are labeled as actually having clinical evidence (4.9%) in supporting their use or benefit, while the vast majority of apps targeting autism (95.1%) offer no clear direct or indirect evidence. By comparing the “autism apps” developed in foreign countries and in mainland China, statistics based on 500 “autism apps” listed on Autism Speaks website report that apps developed in foreign countries mostly focus on enhancing the rehabilitation training of autistic children. In contrast, apps developed in mainland China mostly aim to raise the awareness of ASD among the parents of autistic children [25]. Furthermore, even for apps developed in foreign countries, most of these focus on enhancing the language and behavior abilities of autistic patients, rather than their social abilities such as recognizing or expressing their emotions [25].

As for DVDs, Baron-Cohen and his research team created a children's animation series, called The Transporters (http://www.thetransporters.com) in a highly predictable fashion with lawful and systemizable patterns, which graft real-life faces of actors showing emotions, and contextualized them in entertaining social interactions between a whole family of eight different toy vehicles running on tracks or cables, which have limited degrees of freedom of motion: two trams, two cable cars, a chain ferry, a coach, a funicular railway, and a tractor [26]. The series consists of fifteen 5-min episodes, each of which focuses on a key emotion or mental state. The 15 key emotions include Ekman's Big Six (i.e., happy, sad, angry, afraid, disgusted, and surprised) [27], emotions that are more “complex” but still developmentally appropriate (i.e., sorry, jealous, proud, and ashamed), and emotions and mental states that are important for everyday social functioning (i.e., kind, unfriendly, excited, tired, and joking). Furthermore, a study investigating the effectiveness of individual use of The Transporters animated series (with parental support) over a 4-week period shows that using The Transporters significantly improves levels of emotion comprehension and recognition in 4–7 years old children with ASC in as few as 4 weeks [26]. Baron-Cohen argues that according to the empathizing-systemizing (E-S) theory [14], the reason why children with ASC pick up crucial information in The Transporter about emotional expressions through repetitive watching is that children with ASC can be satisfied with the need for sameness by using average or even above-average systemizing of the motions in The Transporters as they are strongly driven to discover the ***truth*** (i.e., precise, reliable, consistent, or lawful patterns or structure in data).

In 2018, a Chinese research group conducts an intervention study with 14 children (12 boys and 2 girls) with a formal diagnosis of ASD and 7 typically developing children and finds that using animated vehicles with real emotional faces (“The Transporters animated series” are used as the basis for the intervention materials, which are translated into Chinese with some adaptation according to Chinese culture and language) is significantly beneficial to emotion comprehension and recognition skills for Chinese children as well. Their findings also suggest that the DVD is an effective early intervention for Chinese children.

In this paper, we propose a novel framework for human-computer/robot interaction based on a Facial Emotion Cognition and Training System (FECTS) applying the E-S theory and introduce a preliminary intervention study to improve the emotion recognition of Chinese children with ASD and to reduce the rehabilitation workload and mental pressure of the therapists and parents of autistic children. Specifically, the present study contributes to the literature in several important ways.

1. Unlike textbooks or DVDs, our FECTS is a program (i.e., a user interface or an app) that can run on different devices such as mobile phones, intelligent terminals, and display screens of robots. According to the E-S theory, highly structured information processing systems such as mobile devices, computers, and robots, are more likely to be understood and accepted by people with ASD. Therefore, our FECTS has the advantage of pervasive/ubiquitous computing, for example, being accessible at any time, any place with different devices.
2. Our FECTS is designed and developed based on the textbook “Theory of mind IV: Teaching children with autism spectrum disorders emotions (in Chinese)” [19] and constructive suggestions given by experts in the autism domain, resulting in that our FECTS provides children with ASD with practical easy-to-follow graded emotion training contents (e.g., emotion matching training, emotion inference training, and emotion imitation training in a step-by-step order). Thus, our FECTS has guidelines for emotion training in a scientific framework.
3. Unlike most of the “autism apps” developed in mainland China targeting users such as the therapists or parents of Chinese children with ASD, our FECTS targets Chinese children with ASD directly. Consequently, based on the design principles of “The Transporters animated series” [26], we developed a user interface that can attract the interest of autistic children and facilitate their operations in an easy way.
4. Unlike most of the “autism apps” developed worldwide without tracking the learning process of autistic children, we deploy deep-learning algorithms of facial expression recognition and attention analysis in the back end (e.g., devices such as a PC, a robotic system, or a cloud system) implementing our FECTS, which can perform real-time tracking of the imitation quality and attention of the autistic children during the expression imitation phase. By doing so, therapists and parents of Chinese autistic children can track and observe the medical condition as well as emotion learning progress of each autistic child for a long period of time through a back-end data visualization system.
5. Unlike most of the “autism apps” developed worldwide with limited interactive data to analyze, the massive data of the Chinese autistic children interacting with our FECTS can be stored in a cloud system. Combined with the expert's inputs from some rating scales such as the childhood autism rating scale (CARS) [28], the cloud data is then fed into a cloud-based evaluation system to evaluate the emotion learning progress of each autistic child more comprehensively and objectively.

The rest of the paper is organized as follows.  Section 2 introduces a framework for human-computer/robot interaction based on the Facial Emotion Cognition and Training System (FECTS), and the design and implementation of the FECTS. This section also describes deep learning algorithms of facial expressions recognition and attention analysis deployed in the back end of our FECTS. A cloud-based evaluation system with data statistics and visualization that can evaluate the emotion learning progress of each autistic child is also introduced in this section.  Section 3 presents a preliminary study on Chinese autistic children with FECTS as well as results and a detailed discussion. Finally, the conclusions, limitations, and future work are given in Section 4.

## 2. Materials and Methods

### 2.1. Human-Computer/Robot Interaction Framework

**Figure 1:**
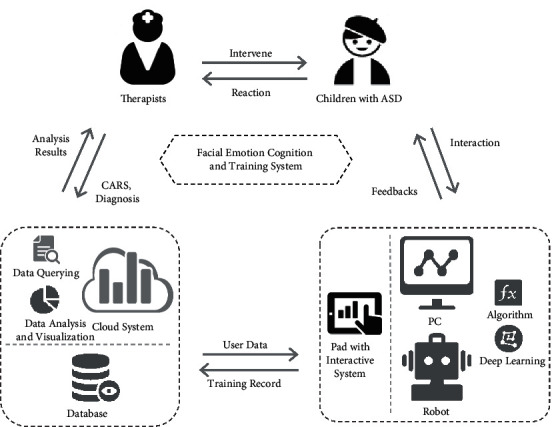
A framework for human-computer/robot interaction based on the Facial Emotion Cognition and Training System (FECTS).

As mentioned earlier, highly structured information processing systems, for example, mobile devices, computers, and robots, are more likely to be understood and accepted by people with ASD. Therefore, to enable Chinese autistic children to access our FECTS at any time and place using different devices, we design a framework (see Figure 1) for human-computer/robot interaction based on our FECTS.

**Figure 2:**
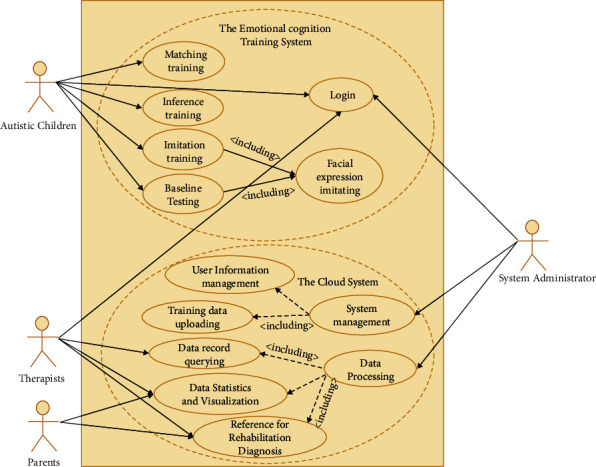
Interaction between the autistic children, the therapists or parents of the children, and the system administrators.

Given below are the principles in designing our framework:

1. The core of the framework is the Facial Emotion Cognition and Training System (FECTS), which can be implemented on PCs, mobile devices such as PADs and smartphones, and robots. This allows Chinese autistic children to access our FECTS with ease. When interacting with these devices, Chinese autistic children can obtain meaningful audio and visual feedback on these devices easily and interactively.
2. The training record (e.g., the tracked record of emotion imitation of the Chinese autistic children interacting with the device implementing our FECTS) is uploaded to and stored in the database of a cloud-based evaluation system. The cloud system feeds the device implementing our FECTS with user data (e.g., patient history) so that the device can provide more appropriate feedback to the Chinese autistic children.
3. Therapists or parents of Chinese autistic children can access the analysis of emotion learning progress of the children through data querying, statistics, and data visualization of the cloud-based evaluation system. The cloud system can receive the experts' or parents' inputs from some rating scales such as CARS or diagnosis of the children.
4. Deep learning algorithms of facial expressions recognition and attention analysis can be deployed in the back end (e.g., devices such as a PC, a robotic system, or a cloud system) implementing our FECTS, which can perform real-time tracking of the imitation quality and attention of the autistic children during the expression imitation phase.
5. The devices implementing our FECTS are mainly for autistic children. Sometimes, their parents can also go through the training process via their devices to provide feedback on improving the design of our FECTS. The cloud-based evaluation system is mainly for the therapists or parents of autistic children for accessing the statistics and data visualization of the emotion learning progress of the children. It is also useful to therapists for improving the rehabilitation training plan by referring to the analysis results and relevant medical data (such as the history of children, CARS, etc.) and for parents to conduct effective support work for the training. By using the cloud system, system administrators can classify, analyze, and visualize a large amount of abstract data and present them in an intuitive form for therapists and parents, making them aware of the emotion learning progress of the children. Refer to Figure 2 for the interaction between the autistic children, the therapists or parents of the children, and the system administrators.

This framework confers many advantages. For instance, the framework ensures a closed-loop interaction (Figure 2) with response and feedback between autistic children, the devices implementing our FECTS, the cloud-based evaluation system, as well as the therapists and parents of the children. As a result, after repeated cycles of interaction, our FECTS receive substantial iterative development, and the correct interactive operations of autistic children are gradually strengthened. Furthermore, the framework takes massive interactive data and the patient history into account to evaluate the emotion learning progress of each autistic child more comprehensively and objectively.

### 2.2. Design and Implementation of Emotional Cognition Training System

#### 2.2.1. Paradigm Design

In this paper, the design of emotional cognition rehabilitation is based on “Theory of mind IV: Teaching children with autism spectrum disorders emotions (in Chinese)” [19]. It is further optimized based on the recommendations of doctors from the Shenzhen Maternal and Child Health Hospital and therapists from the Smart Children Education Center (Shenzhen).

**Figure 3:**
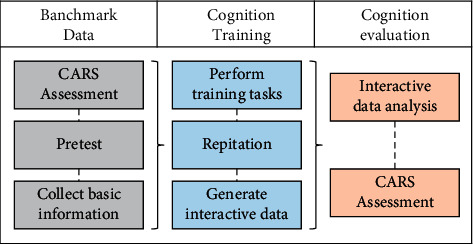
Paradigm design of emotional cognition training.

The training paradigm (Figure 3) consists of three components: (1) acquisition of baseline data: before rehabilitation training, the basic information (gender, age, medical history, etc.) about the children with ASD is recorded. In the baseline test part, the childhood autism rating scale (CARS) is applied to evaluate emotional cognition abilities for children with ASD. (2) Emotional cognition intervention training for children with ASD: according to the guidance in the system, the emotional cognition training is completed step by step. The children are allowed to proceed to the next step after completing the current task. If a task is not complete, the previous training needs to be repeated until the current task is finished. (3) Emotional cognition ability assessment: according to the performance of interaction, their emotional cognition abilities are evaluated. The ability levels are evaluated by the performance of training, the result of facial expression recognition, and the record of interaction performance. If the children with ASD have received long-term rehabilitation training, the CARS is utilized to reassess the improvement of their emotional cognition abilities.

#### 2.2.2. Systematic Design

**Figure 4:**
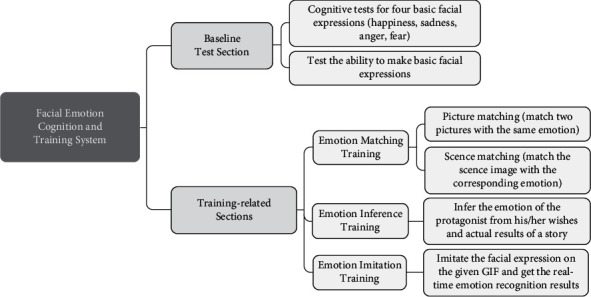
Systematic design of the emotional cognition training system.

The system is designed and developed in strict compliance with the above paradigm (Figure 3). The training content is electronically integrated as a mobile application on Android 5.1. The training tasks are presented in the form of mini games, so the children with ASD may complete the training tasks by simply tapping on the user interface. The system is composed of two sections (see Figure 4): (a) baseline test and (b) training-related sections including emotion matching training, emotion inference training, and emotion imitation training.

**Figure 5:**
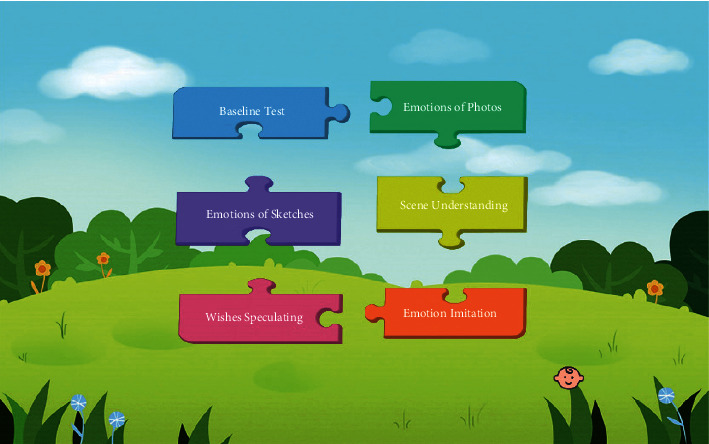
The main interface of our FECTS: Baseline Test is the beginning of the training, followed by Emotions of Photos, Emotions of Sketches, Scene Understanding, Wishes Speculating, and Emotion Imitation.

The main interface of our FECTS consists of six sections (see Figure 5, note that the system was originally designed in Chinese, and we translate it into English for the convenience of the readers of this paper). Originally, these six sections are graded in order of difficulty; thus, it is highly recommended that autistic children should be trained in such order. However, each section can be trained alone if necessary. The content of the six sections of the entire system is designed based on “Theory of mind IV: Teaching children with autism spectrum disorders emotions (in Chinese)” [19]. The training content in the textbook is widely used in the rehabilitation and clinical treatment of ASD children in Hong Kong. For example, ELCHK Hung Hom Lutheran Primary School in Hong Kong used the textbook to train autistic children to put on enough clothes in winter.

#### 2.2.3. The Baseline Test Section

The Baseline Test is a module that evaluates the basic emotional cognition level of a person with autism. It records the initial state of the person before training. Parents are also allowed to participate in this module to experience the basic operation mode of this system.

**Figure 6:**
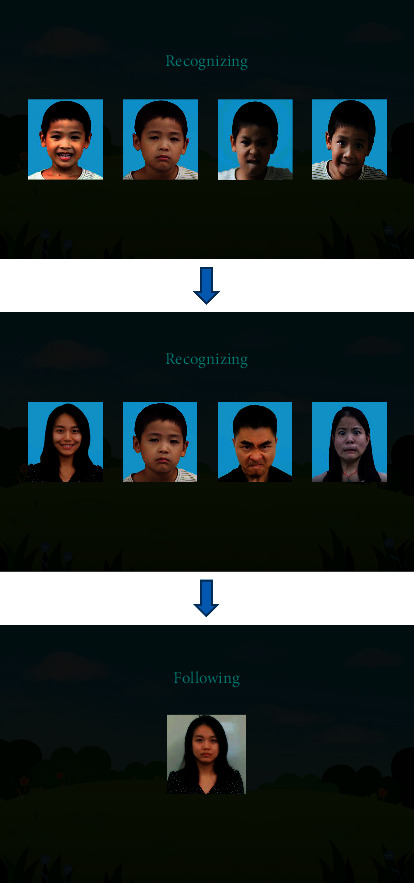
Training that the autistic children should follow in the Pretest section.

When the Baseline Test module is in operation, the system automatically records a video and plays an audio guide. According to the instruction, the child with autism is required to recognize the four most basic emotions (i.e., happiness, sadness, anger, and fear). The child first identifies four different expressions on the same person and then four different expressions on different people. Subsequently, the system requires the child to make these four expressions by referring to the provided expression GIFs (see Figure 6). Since this section is completed entirely by the autistic child, the system records his or her initial ability to recognize and display these four basic emotions.

#### 2.2.4. The Training-Related Sections

Three modules are available in the training-related sections, that is, Emotion Matching Training, Emotion Inference Training and Emotion Imitation Training, which become progressively difficult.

**Figure 7:**
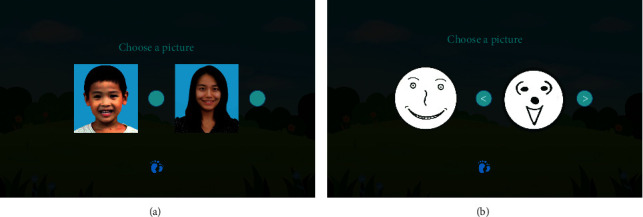
Emotions of Photos (a) and Emotions of Sketches (b) for emotion matching training.

There are two sessions in the Emotion Matching Training module, Emotions of Photos (Figure 7(a)) and Emotions of Sketches (Figure 7(b)). The two sessions are similar in principle but require different materials. Due to the difference in recognition between real photos and abstract cartoons for children with autism, we set the material of Emotions of Photos to be real emotion photos and set the material of Emotions of Sketches to be cartoon facial expressions. As such, they are two separate sessions. In the Emotions of Photos session, the autistic child learns the features of each facial expression photo. After that, s/he is required to recognize the picture that is consistent with the target emotion photo. The Emotion Matching Training is a relatively mechanical and systematic cognitive process, so it is a relatively simple module for a person with autism who has a strong visual memory and an ability to recite objects from memory. The Emotions of Sketches session follows the same process as the Emotions of Photos but utilizes cartoon faces as the material for training.

**Figure 8:**
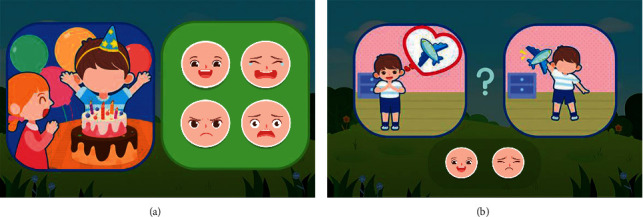
Scene Understanding (a) and Wishes Speculating (b) for emotion inference training.

Compared to the Emotion Matching Training module, Emotion Inference Training is more difficult since at this stage; the child with autism needs to comprehend a given situation and infer associated emotions based on the description of this situation. The Emotion Inference Training module consists of two sessions, Scene Understanding (Figure 8(a)) and Wishes Speculating (Figure 8(b)). In the Scene Understanding session, the child is presented with a description of a situation and has to identify the expected emotion of the protagonist in that situation. Completion of this session requires the autistic child to understand and empathize with the situation. In the Wishes Speculating session, the system describes a short story in which the protagonist makes a certain wish, and the ending of the story is related to this wish. There are two situations, wish-fulfillment and failure to fulfill the wish. According to the protagonist's wish and the ending of the story, the child is required to select the emotion of the protagonist.

**Figure 9:**
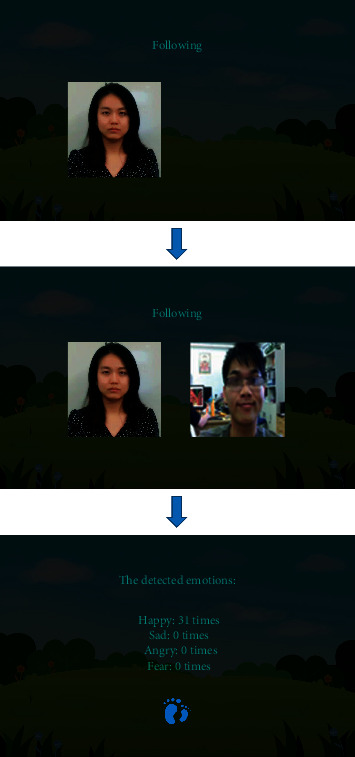
Training steps in the Emotion Imitation Training section.

The final module, Emotion Imitation Training (Figure 9), is the most difficult of all. The therapist needs to supervise and guide the child with ASD. The child is required to make facial expressions along with the provided GIFs. At the same time, the expressions recorded by the camera are displayed on the screen in real time. The screen is equivalent to a mirror for observing his or her current facial expressions. When the facial expressions are imitated correctly, the therapist can provide positive feedback to the child for establishing and reinforcing the correct facial expression mapping. After completing each expression imitation, the screen displays the imitation results based on our expression recognition algorithm. This module has a high-level requirement in terms of coordination and requires the child to demonstrate the abilities of simple generalization and expression imitation.

Since stimuli can have an important effect on children's performance on such tasks, further discussion on the choice and source of stimuli used will be warranted. In the Chinese autism rehabilitation textbook mentioned above, sketched facial expressions, cartoons of facial expressions and scenes, pictures of real people, and so on are used as stimuli to train autistic children in Hong Kong. Since the Chinese autism rehabilitation textbook was chosen as the evaluation standard for designing the FECTS system, similar stimuli were chosen in the FECTS system. However, to make the FECTS system more relevant to autistic children in mainland China, some modifications to the sketches and the cartoons were carried out, and certain pictures of a real person in the textbook were replaced with more appropriate ones. Such modifications were approved by the doctors and autistic professionals in the rehabilitation institutions, such as Shenzhen Maternal and Child Health Hospital in China.

### 2.3. Deep Learning Algorithms of Facial Expressions Recognition and Attention Analysis

#### 2.3.1. Algorithm of Facial Expressions Recognition

**Figure 10:**
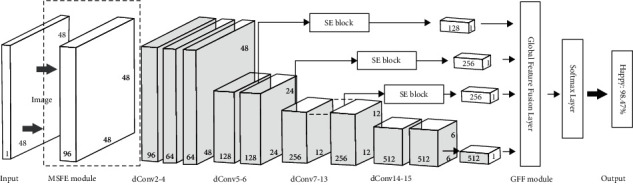
Our proposed CNN architecture based on the MSFE module and the GFF module. Input is the original gray-scale images with 48 × 48 × 1. We omit the arrows in the main structure of the network.

Recently, deep-learning models especially the convolutional neural network (CNN) have demonstrated excellent performance in image classification. It has been widely applied to human facial expression recognition and achieved good results in healthcare applications [28, 29]. In this paper, we propose an algorithm based on deep learning so-called DeepLook, which is used for facial expression recognition in the emotion imitation phase of the training process. The DeepLook algorithm has two characteristics, that is, lightweight network and high precision network. The network structure is shown in Figure 10.

*(1) Lightweight Network*. C. Szegedy et al. [30] propose GoogLeNet with Inception module, which achieves the state-of-the-art for classification and detection in the ImageNet Large-Scale Visual Recognition Challenge 2014 (ILSVRC2014). The inception module demonstrates that the performance of dense components can approximately approach the optimal local sparse connection. Furthermore, by considering that images have different size features, we present a multi-branch architecture named the MSFE module that allows the network to make full use of the information of the original pixels. Inspired by the positive results of MobileNet [31], the use of deep separable convolutions is a good balance between accuracy and computation time. Therefore, we reduce the computational complexity using deeply separable convolution rather than standard convolution in the DeepLook network structure.

*(2) High Precision Network*. W. Liuwe et al. [32] propose an efficient object detector called single-shot multi-box detector (SSD), which adds additional convolution layers to the end of the base network. These layers have decreasing sizes for multi-scale features detection. Inspired by it, we reuse the feature maps of different depths in the network, instead of adding additional convolution layers. We use the feature maps that have been generated. Furthermore, the Squeeze-and-Excitation (SE) blocks in SENet [33] effectively improve the performance of the network with little additional computational cost, allowing the network to learn the interdependencies of each channel of the feature map.

We perform FER experiments on Facial Expression Recognition Challenge 2013 (FER2013) data set to evaluate our proposed method.

Table 1 gives the testing accuracy of our proposed approach and other FER methods on FER2013 data set. Y. Tang [35] achieves the best results with the RBM algorithm in the FER2013 competition. Obviously, our method achieves good performance on the FER2013 data set.

**Figure 11:**
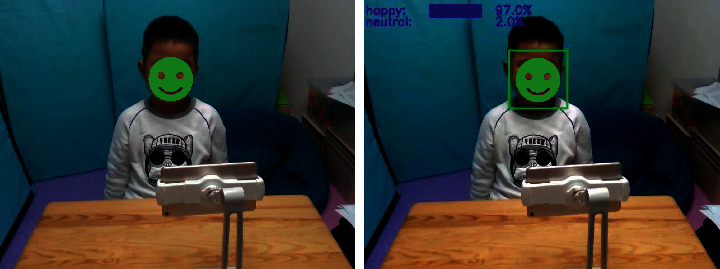
Illustration of the emotion analysis in a child with ASD (green box represents the face location, and blue texts represent predicted emotion labels and probability values).

Since the DeepLook network structure is lightweight and highly precise, it can be used to detect facial expressions (see Figure 11) in real time, returning the detected expression results in real time to the front-end emotional training system. In addition, DeepLook is used to analyze the video of autistic children interacting with our FECTS to obtain the corresponding expression recognition data file, which includes the frame number of each frame in the analysis video, the time stamp, the face detection success flag, and the predicted probability values of the seven expressions. Using the back-end data visualization system described in the next section, we can visualize the expression recognition data files to observe and analyze the emotional state and trends of autistic children interacting with our FECTS.

#### 2.3.2. Algorithm of Attention Analysis

**Figure 12:**
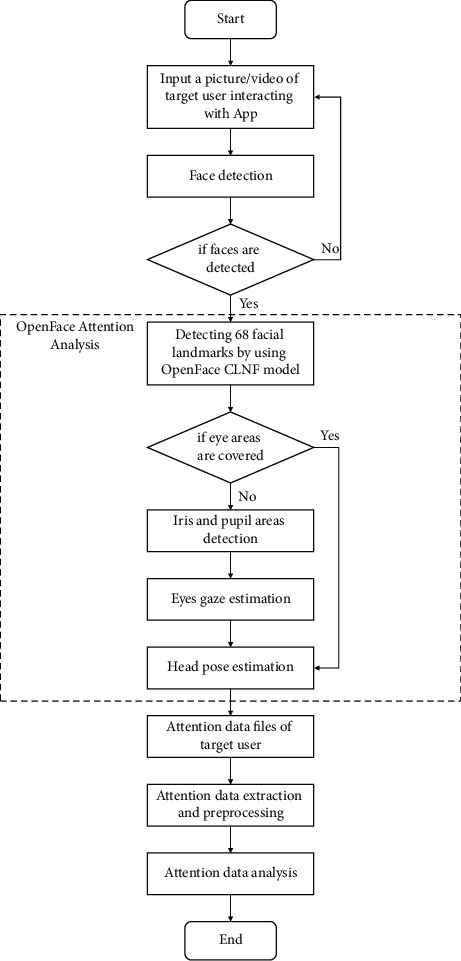
The pipeline of attention analysis by OpenFace.

In the attention analysis of the video of the interaction between autistic children and our FECTS, we use OpenFace [41], an open-source face behavior analysis tool based on deep learning technology. This tool can be used for facial landmark detection, head pose estimation, eye gaze estimation, and facial action unit detection. In this paper, we mainly use the first three functions of OpenFace to realize attention recognition of autistic children. The whole attention recognition process is divided into three parts: face detection, OpenFace attention analysis, and attention data analysis (Figure 12).

*(1) Face Detection*. For each frame of the input video, we must first perform face detection. If the face of an autistic child is detected, the face area can be sent to OpenFace for attention analysis. If the face of an autistic child cannot be detected, we need to skip the attention analysis of this frame of the image and then perform face detection for the next frame.

**Figure 13:**
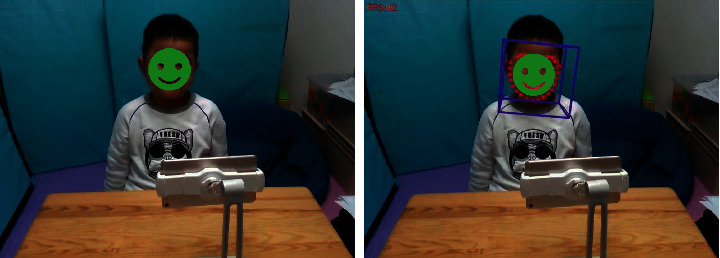
Illustration of the attention analysis for a child with ASD (red dots indicate facial feature points, the blue box indicates estimated head posture direction, and the green line indicates estimated eye gaze direction).

*(2) OpenFace Attention Analysis*. After obtaining the detected face region of children with autism, we use facial feature detection in OpenFace trained on LFPW [37], Helen [42], and Multi-PIE numbers [43] data sets. The model can detect 68 facial feature points in the face region. Through the position of the 68 facial feature points and the relative position and parameters of the camera, the head pose estimation can be performed, and the three rotation angles of the head of the autistic child relative to the camera position are calculated (raw, pitch, and yaw). When the eyes of the face are not covered, we use the CLNF [44] eye feature point detection model trained in the SynthesEyes [45] data set in OpenFace. We further detect 16 irises and 16 pupil features in eye area points. Similarly, based on the iris feature points and pupil feature points and relative position and parameters of the camera, eye line of sight estimation can be performed (Figure 13), and two rotation angles (pitch and yaw) of the eyeball of the autistic child relative to the camera position are also calculated.

*(3) Attention Data Analysis*. After OpenFace attention analysis, we obtain a data file containing the attention information of children with autism, including the frame ID of each frame in the analysis video, time stamp, face detection confidence, face detection succeed flag, 68 face feature points 2D position, 56 eye feature points 2D position, 2 eyes gaze estimation angles, and 3 head posture estimation angles (Figure 13). We use the estimated head posture yaw angle and the yaw angle of the eyes gaze to average the attention direction of the child in the horizontal direction of the video. At the same time, we also use the head posture pitch angle and the pitch angle of the eyes gaze to calculate the attention direction of the child in the vertical direction of the autism.

Using the back-end data visualization system described in the next section, we visualize the attention-aware data files to observe and analyze the attention states and trends of autistic children interacting with our FECTS.

### 2.4. The Cloud-Based Evaluation System

#### 2.4.1. A Cloud System

**Figure 14:**
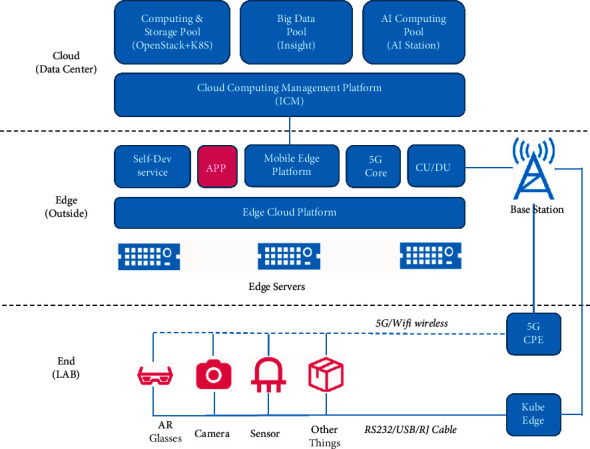
AIRS cloud architecture based on Inspur server cluster.

The Research Center for Industrial Internet of Things (SCIoT Center) is a large-scale industrial cloud and data center established by the Shenzhen Institute of Artificial Intelligence and Robotics for Society (AIRS). The center provides a common research and development platform for a variety of areas including cloud computing, big data processing, artificial intelligence, and cognitive computing through a server cluster containing both Tesla V100 and 2080Ti GPUs (Figure 14).

#### 2.4.2. Interaction between Users and Data

We build a cloud-based assessment and diagnostic system based on AIRS cloud architecture that supports logging on by the system administrator, parents, and therapist at anytime and anywhere. When a child with ASD completes the emotional cognition training, the raw data generated in the training is uploaded to the cloud-based system. The cloud-based system is capable of providing three levels of data processing services: data record querying, data statistics and visualization, and data analysis-based rehabilitation diagnosis.

The cloud-based system authenticates the user identity and provides specific services according to different levels of user authorization. The raw data of the emotional cognition training are imported and updated by the system administrator and subsequently provided to the therapist. The statistical and visualized results are available to the therapist and parent. The therapist utilizes the data analysis results to record rehabilitation diagnoses for all users and ultimately feed back into the training process of the child with ASD for treatment improvement and enhancement.

#### 2.4.3. Data Record Querying

Detailed raw data consist of recordings of every training session for every child with ASD who participated in the emotional cognition training and are stored in a database on our cloud-based system. These data exist in two forms: (1) training data generated by the training system and stored as text and numerical values and (2) video data collected by cameras set up in the training area and stored as binary data. Accordingly, the data processing section of the cloud-based system is divided into training data and video data. Each record in the training data contains the ASD child's identity information, training time, training items, answer results, and so on. Each record in the video data contains images of the ASD child in the training process, and it reflects the change in the child's emotion and attention.

**Figure 15:**
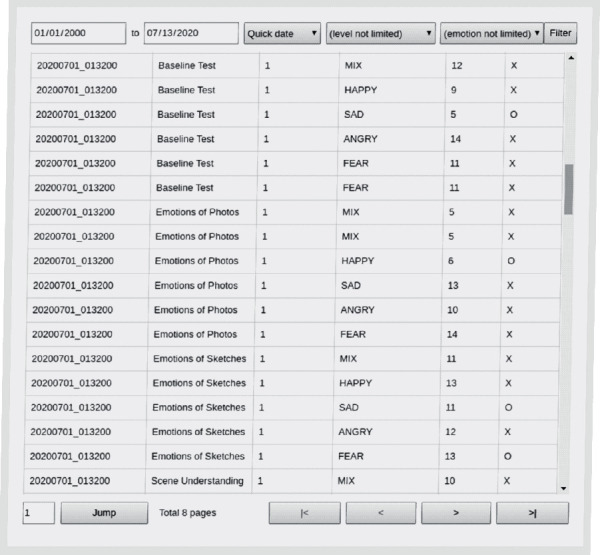
An example of a data querying interface of the cloud system.

The cloud-based system provides a well-interactive data query interface (Figure 15). When users log into the system, they have corresponding permissions to query record entries of specific training data or video data from the database in the back end of the cloud-based system by specifying one or more combinations such as any child with ASD, any phase of time, and any training item as filtering conditions.

These data are raw data generated and recorded during the training process and are used only for querying and subsequent statistical analysis without any modification or deletion. In the presence of any abnormal result, it is possible to backtrack and analyze the corresponding raw data.

#### 2.4.4. Statistics and Visualization

In the cloud-based system, the statistics and visualization are further data processing methods based on the data record querying. For each child with ASD who participated in this emotional cognition training, each of his or her data records can be represented in three different dimensions, training time, training item, and diagnostic reference item. The training time is divided by the basic unit, that is, days, and it can be extended to weeks, months, and years as deemed appropriate. The training item is the training processes conducted on mobile devices, including Pre-Testing, Emotion Matching Training, Emotion Inference Training, Emotion Imitation Training, and so on. The diagnostic reference item is the data representing the autistic child's training performance, which can be divided into two categories, that is, emotion types such as happy, sad, angry, and fear for emotion types and attention directions including eyes yaw, head yaw, eyes pitch, and head pitch. By selecting any combination of the three different dimensions, our cloud-based system can automatically draw visualized figures to conveniently and accurately represent the improvement of training for each child with ASD.

**Figure 16:**
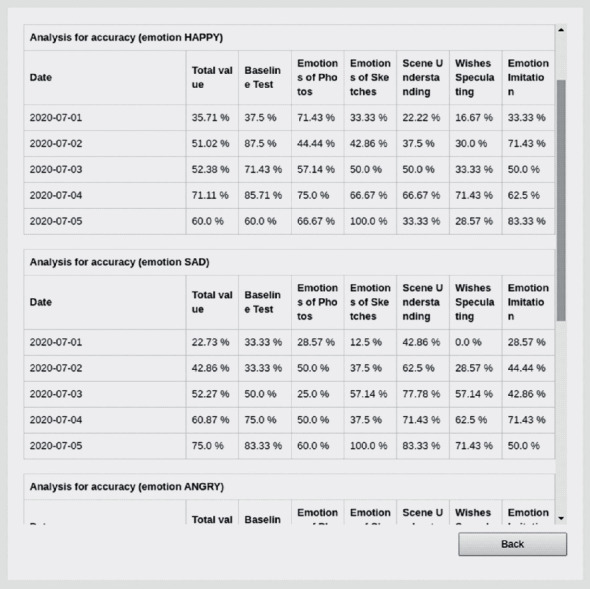
An example of a statistical tabular interface of the training data module.

With the interactive training data, for any combination of the three different dimensions, there are three different metric parameters: the number of training records, the percentage of correct answers, and the average training time. These parameters are calculated based on the original data records in one or more dimensions. The results are summarized in a table (Figure 16) that contains the statistical information of a given child with ASD in an emotional cognition training process. Furthermore, the data for secondary analysis, such as the number of training sessions attended, the average accuracy of answers, and the average time spent on each round of training, can be conveniently extracted from this table.

**Figure 17:**
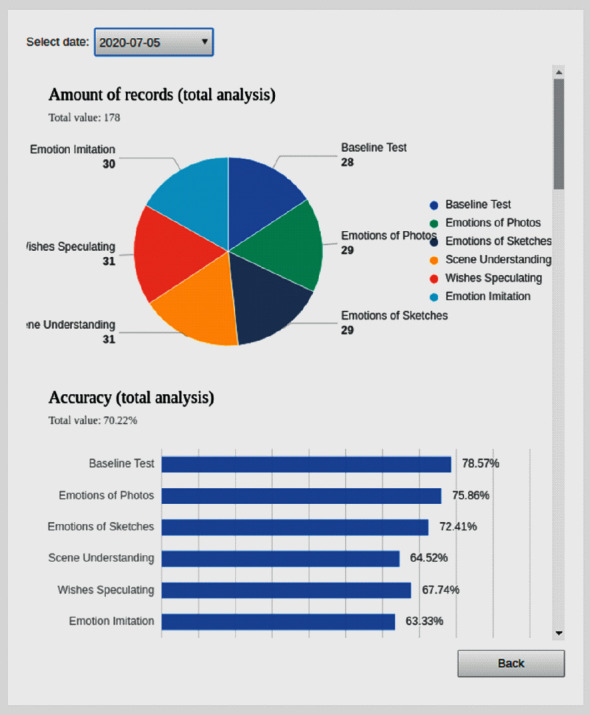
An example of a visual graphical interface of the training data module.

Various types of figures can be generated by selecting the statistical items from the three metric parameters. The figures (e.g., Figure 17) demonstrate how the training effects of children with ASD change over time and across items during emotional cognition training. In the dimension of the training time, different time scales illustrate whether and to what extent the training effects of children with ASD have been improved. In the dimension of training items, the results of item selections demonstrate the corresponding training performance in different item difficulties. In the dimension of emotion types, the results reflect the performance of training and recognition in a specific emotion, and how much improvement is achieved in different emotion cognition processes. Through these types of analyses, therapists and parents can directly observe the extent of the children's cognition improvement.

#### 2.4.5. Data Analysis for Therapists

Based on statistics and visualization, further rehabilitation diagnosis can be analyzed by the cloud-based system. After the completion of phase one of the emotional cognition training, the data is imported and visualized on the cloud-based system. By accessing the cloud-based system, the therapist can obtain statistical information by setting different dimensions and metric parameters. Using the tables and graphical interface, the therapist can evaluate the recovery of the child's condition, analyze the current training performance, and propose improvements to the next stage of emotional cognition training.

The analysis of the training effect and diagnostic comments proposed by the therapist are entered into the cloud-based system. This information is also accessible to the system administrator and parents of the child. Through the cloud-based system, the parents are able to view the effects of rehabilitation in real time based on a statistical analysis of the data and the diagnostic comments provided by the therapist. The system administrator can improve the next phase of the emotional cognition training based on the data records, data analysis results, and diagnostic comments. Therefore, the participants may receive a more customized training plan for rehabilitation.

## 3. Results and Discussion

### 3.1. Procedure

**Figure 18:**
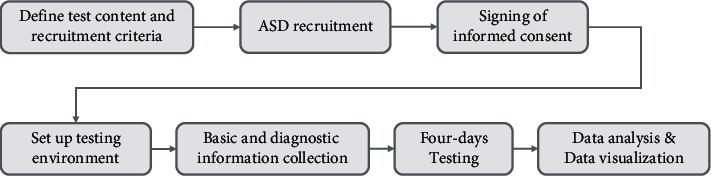
Our preliminary study pipeline.

In this paper, we design a pipeline for our preliminary study (see Figure 18). First, we develop a series of inclusion and exclusion criteria for recruiting participants based on the interaction rules of the emotional cognition and rehabilitation training system. Second, these criteria, along with the test content, test duration, and test purpose, are submitted to Shenzhen Maternal and Child Health Hospital, which assists us recruiting participants and tailoring this information specifically for these autistic children. Third, parents of the children are invited to experience this training system introduced by our developmental team and autism specialists. Parents who agree to their children's participation will sign informed consent forms. Fourth, after obtaining parents' permission, we develop a testing environment, including site selection and facility preparation. Fifth, the basic information and medical records of the children are entered into the training system for reference. Finally, the Pre-Testing and training sessions last at least three days. The daily training lasts approximately 20 minutes with the same content as emotional cognition training.

### 3.2. Test Setup and Participants

According to the experimental aims, we develop an appropriate environment and a series of criteria for participants. The criteria of selection are children (1) with diagnosed ASD, (2) between 5 and 10 years of age, (3) with oral comprehension and presentation skills, and (4) in good health status. The conflicts to the selection criteria are children with (1) other mental disorders, (2) other physical illnesses that conflict with the test, and (3) suspected of or diagnosed with hearing or visual impairment. Based on these criteria, we recruit 12 children from the Shenzhen Maternal and Child Health Hospital (SMCHH) to take the test. In the end, 10 children attend the test; 2 children withdraw due to family reasons and scheduling problems.

**Figure 19:**
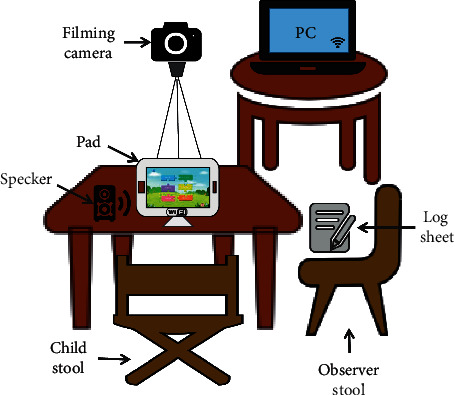
Our preliminary study setup.

To provide a relaxed and suitable environment for the children with ASD, we select a commonly used classroom as the testing site in the Children Rehabilitation Center of Shenzhen Maternal and Child Health Hospital. This room consists of two chairs, a table, a kit of devices for emotion recognition and rehabilitation training, and some interaction log sheets (see Figure 19). Specifically, the kit is composed of a PAD with a training system, a PC with an analysis system, a mini Bluetooth speaker, and a D435 camera.

### 3.3. Data Collection

During the testing period, we collect the following data: (1) training data recorded by the system, (2) video data of facial expressions and attention recorded by the camera, and (3) an interaction sheet manually recorded by the observer.

After four days of testing, the system records a total of 864 training data generated by 10 autistic children who actually complete the entire test. The 540 out of 864 training data are valid, including 154 emotion recognition results. Meanwhile, a total of 133 video data (71 valid data) are obtained, with a total duration of 330 minutes and an average duration of 150 seconds per video file.

### 3.4. Results with Data Analysis and Visualization

After combining the training data recorded by the system and the manually recorded interaction sheets, we conduct user-based analysis and time-based analysis, respectively.

For the user-based analysis, we observe that among the ten children who participate in the test, four children under the age of five often stall at some stage of the training process by repeating the same operation or exiting the system interface due to randomly tapping. These situations are almost interpreted as the inability to perform effective operations, so the data of the four children are not analytically meaningful. For the remaining six children aged between six and eight years, their training data are more complete and analytically meaningful. Five children complete all the training tasks on the last day, and one 5-year-old child does not finish the Emotions of Photos session. Based on our samples, we find that the children under 5-year-old are not able to use the system effectively and train for autonomous rehabilitation.

**Figure 20:**
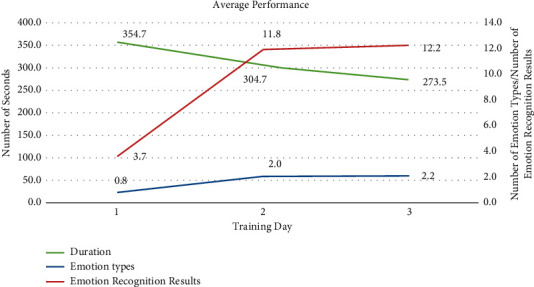
Interaction data results between the FECTS system and manual recordings (*y*-axis values are the average training duration on the left and the number of emotion types and the number of emotion recognition results on the right; *x*-axis values are the training days).

For the time-based analysis, we observe that all six children achieve considerable improvement in training time. Their average training time decreased from 354.7 seconds on the first day to 273.5 seconds on the third day. The average time to complete the equivalent training task on the third day was significantly lower than that on the first day. In the expression imitation session, the average number of recognized emotions and the types of identified emotions on the third day are superior to those on the first day (see Figure 20). Five children achieve 100% completion on the final day of the training task. Four of them achieve this level on the first day, and the other child progressively reaches this level. With respect to giving correct answers, three children improve their accuracy on the third day compared to the first day; two children remain at the same level; and one child experience a decrease.

Overall, it is obvious that in this test most children who are capable of effective training have been able to self-correct. For example, after making the wrong choice for the first time, they are able to select the right answer in the second choice. However, if the second choice is not made immediately, there is a high probability that the wrong choice is repeated. The participants are most likely to confuse sadness with anger. In a few cases, there is confusion between sadness, anger, and fear. The sadness emotion has a high rate of errors in scene recognition, and its corresponding incorrect choice is mostly anger, followed by fear.

In addition, we utilize the DeepLook expression recognition algorithm and the OpenFace attention algorithm to analyze the video of the Emotion Imitation Training session of the front-end emotion cognition and training system. The video analysis results of this session can be applied to assess the emotional cognition and expression abilities of these children with ASD after the first four sessions of the front-end training system.

We mainly concentrate on the video data analysis of the four children who complete the three-day test of our front-end emotion cognition and training APP system. The analyzed results are subsequently imported into the back-end data visualization system for further analysis and visualization.

**Figure 21:**
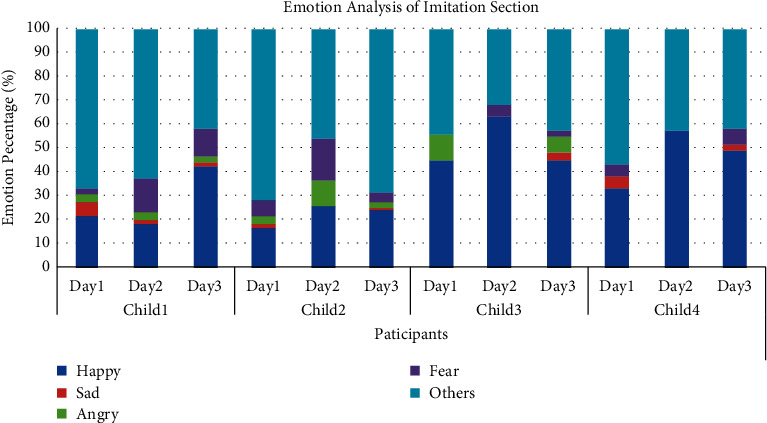
Results of the DeepLook expression recognition (the value on the *y*-axis represents the proportion of that expression occurring in the entire emotion imitation session).

We obtain the frequency of facial expressions during the three-day Emotion Imitation Training session for the four children with ASD (Figure 21). Figure 21 indicates that the proportion of valid expressions (happy, sad, angry, and fear) is notably increased, and the proportion of invalid expressions (others, including neutral, surprise, and disgust) is decreased. This suggests that after three days of training with the front-end emotional cognition and training system, the recognition and imitation abilities of four effective expressions are significantly improved. It is notable that the children with ASD have a weak ability to imitate sad and angry, and a relatively strong ability to imitate happy. Moreover, Child1, Child2, and Child4 have an improved ability to imitate fear after training.

**Figure 22:**
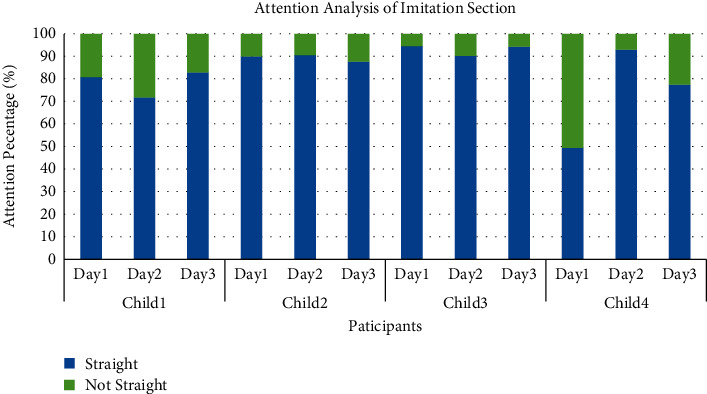
Results of OpenFace attention recognition (the *y*-axis value represents the proportion of that attention direction occurring in the entire emotion imitation session).

Similarly, the frequency of attention directions for the four children with ASD during the three-day emotion imitation session is given in Figure 22. The left, right, and other attention directions are categorized as not straight. The results in Figure 22 demonstrate that the four children with ASD retain their attention most of the time and show great interest in interacting with the front-end emotion cognition and training system.

## 4. Conclusions and Future Work

### 4.1. Conclusions

In this paper, we propose a novel framework for human-computer/robot interaction based on a Facial Emotion Cognition and Training System (FECTS). We also conduct a preliminary intervention study to determine if our FECTS may improve the emotion recognition of Chinese children with ASD. The training data and the interactive data during the preliminary study are all recorded using the FECTS and are uploaded, analyzed, and visualized by a cloud-based evaluation system. We find that among the ten Chinese autistic children, the performance of six subjects has improved in terms of operation proficiency and duration. Five out of six children finish all six training tasks and four out of six children displayed progress in expression imitation. However, four children under the age of five experience difficulty in operating the FECTS. In addition, all ten children showed great interest in the FECTS. Our preliminary results validate the effectiveness of our algorithms of facial expressions recognition and attention analysis and the feasibility of the developed FECTS to some extent.

To sum up, compared to autistic training methods such as card teaching, smartphone apps, and DVDs (e.g., animated cartoons), our FECTS has a number of merits. First, it has the advantage of pervasive/ubiquitous computing, being accessible at any time and any place on different devices. Second, it provides children with ASD with practical easy-to-follow graded emotion training contents that are scientific in nature. Third, it targets the Chinese children with ASD directly with a user interface that can attract the interest of autistic children and facilitate their operation in an easier way. Fourth, therapists and parents can track and observe the illness condition as well as emotion learning progress of each autistic child for a long period of time owing to the deep-learning algorithms of facial expressions recognition and attention analysis in the back end implementing our FECTS. Fifth, the massive interactive data between the Chinese autistic children and our FECTS can be stored in a cloud-based evaluation system, so as to evaluate the emotion learning progress of each autistic child more comprehensively and objectively.

### 4.2. Limitations

There are limitations that hinder the performance of our system.

1. Due to the influence of the lighting and camera position, angle, and other factors at the experimental site, some interaction video files have poor quality, resulting in the decreased performance of the expression recognition and attention recognition algorithms.
2. Our proposed DeepLook expression recognition algorithm does not have a high recognition rate for fear expressions and is prone to misidentify fear as surprise and disgust expressions in practice. There is room for improvement in terms of the robustness and recognition rate of this algorithm.
3. Since the experiments are only for verifying the feasibility of the emotion recognition and rehabilitation training system for children with ASD, a small group of participants is involved. We only demonstrate the proof of concept of the system, but more children with ASD are needed to demonstrate the effectiveness in emotional cognition improvement of our system in further experiments.
4. This program was aimed specifically at children aged between 5 and 10 years. In this study, we recruited 2–3 years old ASD children for a pre-test based on the ASD golden intervention period criteria mentioned in the literature [19]. After our observation and doctor's evaluation, we found that children aged 2–4 neither understand the content of our FECTS system (speech and picture understanding) well nor can they complete the entire training process. Following the advice of doctors, we adjusted the age range of the research targets and changed it to children aged 5–10 to conduct the experiment. Moreover, the textbook written and used in Hong Kong mentioned above stated that the training content is more appropriate for children aged 6–12. Our experiments verified that the FECTS system is well understood and accepted by ASD children over 5 years old, and the difficulty of training is appropriate.
5. The subjects in our study were Chinese children only, which might introduce bias into our work. For instance, our app is in Chinese, and the app was first applied to Chinese autistic children in Shenzhen, China. However, we believe that our FECTS system may be adapted to autistic children from other countries as well. First of all, the Chinese autism rehabilitation textbook chosen for designing the FECTS system was based on theories and contents in the English textbook “Teaching Children with Autism to Mind-read: A Practical Guide for Teachers and Parents” [20], which aims to provide an easy-to-follow graded teaching guide to special needs teachers, educational and clinical psychologists, speech and language therapists, as well as carers of children with autism spectrum conditions. The English textbook mentioned above was highly recommended by the Child Psychology Psychiatry and RCSLT Bulletin in 2000. Thus, our FECTS has guidelines for emotion training in a scientific framework and has the potential for worldwide use. For those without access to the Chinese autism rehabilitation textbook, they may refer to the English textbook to determine if the FECTS system can be applied in a different social/cultural/linguistic context. Furthermore, based on the design principles of “The Transporters animated series” [27], which were significantly beneficial to emotion comprehension and recognition skills for both European and Chinese children, we developed a user interface that can attract the interest of autistic children and facilitate their operations in an easy way. As such, our FECTS system may facilitate children with different cultural backgrounds as well. Nevertheless, to adapt our FECTS system to children from other countries, some scenes and pictures of real people should be replaced under careful consideration of the social/cultural/linguistic context. In addition, UI design that facilitates the operation of the FECTS system would be necessary for such adaptation in other countries.

### 4.3. Future Work

In the future, more standardized clinical studies are needed to further verify the feasibility and effectiveness of the system.

1. To alleviate the interference of camera recording position, angle, and other factors, we may integrate the whole system on a robot and record the interactive video through the fixed camera on its chest.
2. We may improve the recognition rate of fear expressions by the DeepLook algorithm and generate a final output by combining the result of the third-party expression recognition software.
3. We may cooperate with more than three autism rehabilitation institutions and recruit more than 60 autistic children for clinical tests. To reduce the bias in our work, children with different cultural/sociological/linguistic contexts should be recruited in future studies. Meanwhile, the intervention time may be increased to more than one month.

## Figures and Tables

**Table 1:** Testing accuracy for different methods on the FER2013 data set.

Year	Method	Testing accuracy (%)
2013	I. J. Goodfellow et al. [34]	69.26
2013	Y. Tang [35]	71.16
2016	K. Liu et al. [36]	65.03
2016	A. Mollahosseini et al. [37]	66.40
2016	B. K. Kim et al. [38]	70.58
2016	Y. Guo et al. [39]	70.60
2017	T. Chang et al. [40]	73.40
2018	Our proposed approach	74.78

## Data Availability

All data included in this study are available from the corresponding authors upon request.
